# Exogenous and Endogenous Hydrogen Sulfide Protects Gastric Mucosa against the Formation and Time-Dependent Development of Ischemia/Reperfusion-Induced Acute Lesions Progressing into Deeper Ulcerations

**DOI:** 10.3390/molecules22020295

**Published:** 2017-02-15

**Authors:** Marcin Magierowski, Katarzyna Magierowska, Magdalena Hubalewska-Mazgaj, Zbigniew Sliwowski, Robert Pajdo, Grzegorz Ginter, Slawomir Kwiecien, Tomasz Brzozowski

**Affiliations:** 1 Department of Physiology, Jagiellonian University Medical College, 31-531 Cracow, Poland; m.magierowski@uj.edu.pl (M.M.); k.jasnos@interia.pl (K.M.); magdalena.hubalewska@uj.edu.pl (M.H.-M.); agazs@poczta.fm (Z.S.); mppajdo@cyf-kr.edu.pl (R.P.); grzegorz.ginter@uj.edu.pl (G.G.); skwiecien@cm-uj.krakow.pl (S.K.); 2 Department of Genetic Research and Nutrigenomics, Malopolska Centre of Biotechnology, Jagiellonian University, 30-387 Cracow, Poland

**Keywords:** hydrogen sulfide, ischemia/reperfusion, gastric blood flow, prostaglandins, afferent sensory neurons

## Abstract

Hydrogen sulfide (H_2_S) is an endogenous mediator, synthesized from l-cysteine by cystathionine γ-lyase (CSE), cystathionine β-synthase (CBS) or 3-mercaptopyruvate sulfurtransferase (3-MST). The mechanism(s) involved in H_2_S-gastroprotection against ischemia/reperfusion (I/R) lesions and their time-dependent progression into deeper gastric ulcerations have been little studied. We determined the effect of l-cysteine, H_2_S-releasing NaHS or slow H_2_S releasing compound GYY4137 on gastric blood flow (GBF) and gastric lesions induced by 30 min of I followed by 3, 6, 24 and 48 h of R. Role of endogenous prostaglandins (PGs), afferent sensory nerves releasing calcitonin gene-related peptide (CGRP), the gastric expression of hypoxia inducible factor (HIF)-1α and anti-oxidative enzymes were examined. Rats with or without capsaicin deactivation of sensory nerves were pretreated i.g. with vehicle, NaHS (18–180 μmol/kg) GYY4137 (90 μmol/kg) or l-cysteine (0.8–80 μmol/kg) alone or in combination with (1) indomethacin (14 μmol/kg i.p.), SC-560 (14 μmol/kg), celecoxib (26 μmol/kg); (2) capsazepine (13 μmol/kg i.p.); and (3) CGRP (2.5 nmol/kg i.p.). The area of I/R-induced gastric lesions and GBF were measured by planimetry and H_2_-gas clearance, respectively. Expression of mRNA for CSE, CBS, 3-MST, HIF-1α, glutathione peroxidase (GPx)-1, superoxide dismutase (SOD)-2 and sulfide production in gastric mucosa compromised by I/R were determined by real-time PCR and methylene blue method, respectively. NaHS and l-cysteine dose-dependently attenuated I/R-induced lesions while increasing the GBF, similarly to GYY4137 (90 μmol/kg). Capsaicin denervation and capsazepine but not COX-1 and COX-2 inhibitors reduced NaHS- and l-cysteine-induced protection and hyperemia. NaHS increased mRNA expression for SOD-2 and GPx-1 but not that for HIF-1α. NaHS which increased gastric mucosal sulfide release, prevented further progression of acute I/R injury into deeper gastric ulcers at 6, 24 and 48 h of R. We conclude that H_2_S-induced gastroprotection against I/R-injury is due to increase in gastric microcirculation, anti-oxidative properties and afferent sensory nerves activity but independent on endogenous prostaglandins.

## 1. Introduction

Hydrogen sulfide (H_2_S) plays an important role as intracellular gaseous transmitter and contributes to many physiological and pathological processes [1,2]. This molecule exerts vasoactive activity similarly to nitric oxide (NO) and carbon monoxide (CO), all three considered to act as endogenous gaseous mediators [3,4,5,6]. Moreover, H_2_S similarly to NO, inhibits leukocytes adherence to blood vessel wall [7]. Endogenous H_2_S is synthesized from l-cysteine by the activity of two main pirydoxal-5-phosphate (P5P, vitamin B_6_) dependent enzymes: cystathionine γ-lyase (CSE) and cystathionine β-synthase (CBS) [8,9] or by 3-mercaptopyruvate sulfurtransferase (3-MST) in co-activity with cysteine aminotransferase [10].

H_2_S plays an important role in the regulation of the physiological functions of gastrointestinal (GI) tract and the mechanism of GI-integrity maintenance [11]. Physiologically, H_2_S regulates Cl^−^ ion secretion by activating Ca^2+^ and ATP-sensitive K^+^ channels in rat colon [12]. In another study, Ise et al. [13] have demonstrated that NaHS, a H_2_S donor, dose-dependently increased HCO_3_^−^ ion secretion in rats’ small intestine. On the other hand, recent data has shown that H_2_S may be involved in human colon cancer cell proliferation and this mechanism is accompanied by a decrease of NO production [14]. In contrast, it has been reported that diallyl disulfide, a H_2_S donor, dose-dependently inhibited HT29 human colon cancer cells proliferation indicating that these discrepancies are dose related [15]. Lou et al. [16] have shown that H_2_S inhibited the lipid peroxidation and formation of water immersion and restraint stress-induced gastric damage. Wallace et al. [17] observed that treatment with l-cysteine and H_2_S donors accelerated the healing of experimental chronic gastric ulcers. Furthermore, H_2_S has been shown to prevent gastric mucosal damage induced by intragastric application of ethanol, alendronate or non-steroidal anti-inflammatory drugs [6,18,19].

NaHS abrogated the gastric epithelial cells against I/R injury via mitogen-activated protein kinase-dependent anti-apoptotic action and NF-κB-dependent anti-inflammatory pathway [20]. Moreover, NaHS attenuated harmful effect of short term I/R in rat gastric mucosa and this effect was accompanied by upregulated superoxide dismutase (SOD) activity [21]. Mard et al. [22] observed that the systemic (i.v.) administration of NaHS and l-cysteine attenuated gastric injury induced by I/R. Despite these reports concerning the effect of NaHS in the upper and lower GI-tract, the potential role of this gaseous molecule in protection against gastric I/R injury and the mechanism of its protective action has not been fully investigated. Little is known whether this endogenously produced H_2_S can affect gastric lesions caused by I/R with the respect to their progression into deeper gastric erosions caused by extending the time of reperfusion up to 12, 24 or 48 h. Moreover, the potential gastroprotective effect of slowly H_2_S-releasing compound, GYY4137 against I/R-induced gastric damage has not been so far investigated [23]. Furthermore, it remains unknown whether the mechanism of H_2_S-mediated gastroprotection against this type of gastric mucosa injury involves regulation of oxidation and hypoxia, activity of prostaglandins (PGs)/cyclooxygenases (COX) system, afferent sensory nerves releasing calcitonin gene related peptide (CGRP) and vanilloid receptors (TRPV)-1 activity.

## 2. Results

**Figure 1:**
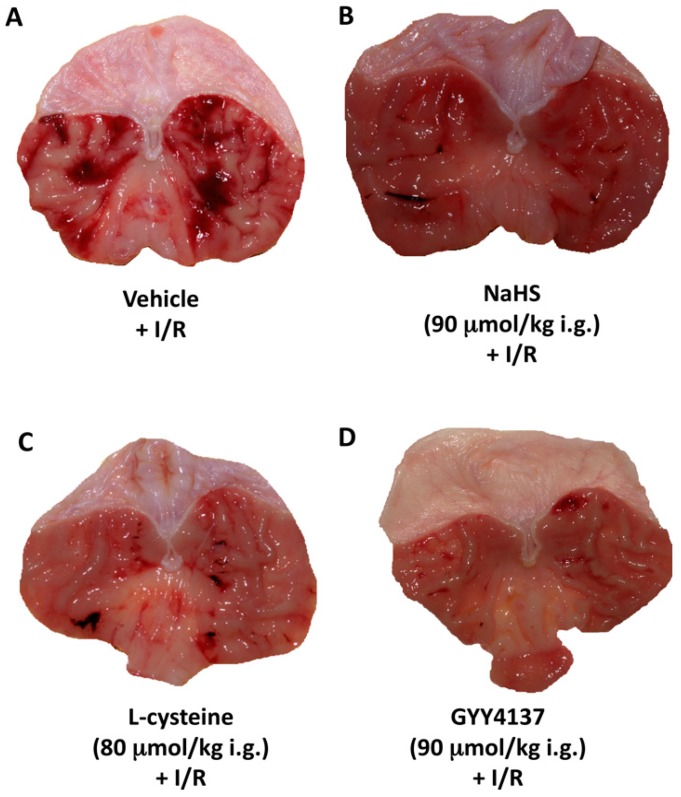
Gross macroscopic appearance of gastric mucosa pretreated 30 min prior to gastric ischemia-reperfusion (I/R) with vehicle (**A**); NaHS (90 μmol/kg i.g.); (**B**), l-cysteine (80 μmol/kg i.g.); (**C**) or GYY4137 (90 μmol/kg i.g.); (**D**) and exposed to 30 min of I of celiac artery followed by 3 h of R (I/R).

Exposure to I followed by 3 h of R caused the extensive hemorrhagic erosions in gastric mucosa of rats pretreated with vehicle-control (Figure 1A). Figure 1B shows that the area of I/R-induced gastric damage is reduced in rats pretreated with NaHS (90 μmol/kg i.g.) as compared with vehicle-control (Figure 1B vs. Figure 1A). Similarly, I/R-induced gastric mucosa damage was attenuated by pretreatment with H_2_S precursor, l-cysteine (Figure 1C vs. Figure 1A). Pretreatment with GYY4137 (90 μmol/kg i.g.) reduced I/R-induced gastric damage formation comparing with vehicle-control (Figure 1D vs. Figure 1A).

**Figure 2:**
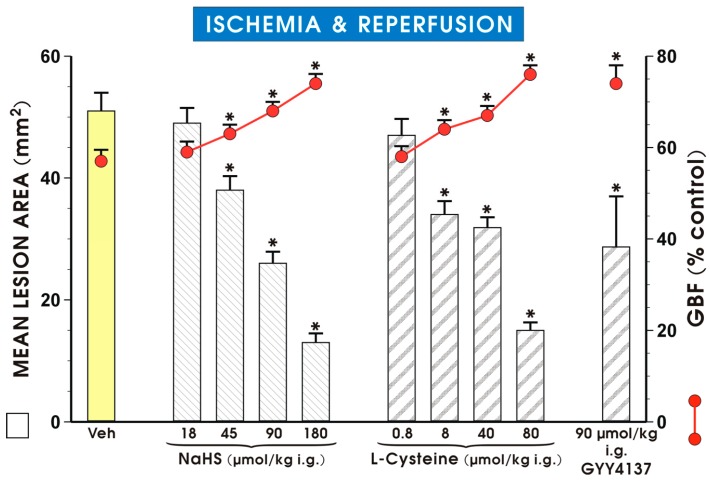
Mean lesion area and the GBF in gastric mucosa of rats pretreated with vehicle, NaHS (18–180 μmol/kg i.g.), l-cysteine (0.8–80 μmol/kg i.g.) or GYY4137 (90 μmol/kg i.g.) and exposed to I followed by 3 h of R. Results are mean ± S.E.M of 4–5 rats per each experimental group. Significant changes as compared with the respective values in the vehicle-control group is indicated by an asterisk (*p* < 0.05).

Pretreatment with NaHS (18–180 μmol/kg i.g.) and l-cysteine (0.8–80 μmol/kg i.g.) dose-dependently attenuated the area of gastric lesion and significantly increased the GBF in rats exposed to I followed by 3 h of R (Figure 2). The dose of NaHS and l-cysteine which reduced by 50% of the mean area of gastric lesion comparing with vehicle-control group was 90 and 80 mg/kg, respectively (*p* < 0.05). Pretreatment with GYY4137 administered i.g. in a dose of 90 μmol/kg, equimolar with the dose of NaHS inhibiting I/R-induced lesions by 50%, significantly decreased the area of I/R-induced gastric damage and significantly raised the GBF as compared with vehicle-control group (*p* < 0.05) (Figure 2).

**Figure 3:**
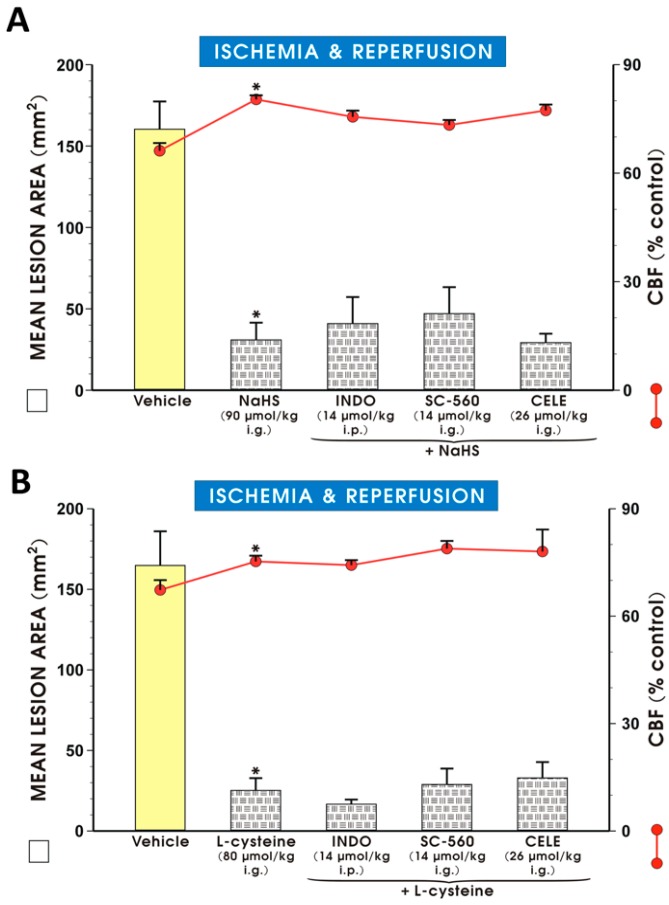
Mean lesion area and GBF in gastric mucosa of rats pretreated with vehicle, NaHS (**A**) or l-cysteine (**B**) alone or in combination with indomethacin (INDO, 14 μmol/kg i.g.), SC-560 (14 μmol/kg i.p.) or celecoxib (CELE, 26 μmol/kg i.g.) and exposed to I followed by 3 h of R. Results are mean ± S.E.M of 4–5 rats per each experimental group. A significant change as compared with the respective values in the vehicle-control is indicated by asterisks (*p* < 0.05).

Figure 3 shows that co-administration of NaHS (90 μmol/kg i.g.) or l-cysteine (80 μmol/kg i.g.) with 1) non-selective COX inhibitor, indomethacin (14 μmol/kg i.p.), 2) selective COX-1 inhibitor, SC-560 (14 μmol/kg i.g.) or 3) selective COX-2 inhibitor, celecoxib (26 μmol/kg i.g.) (Figure 3A,B, respectively) remained without a significant influence on the NaHS- and l-cysteine-induced decrease in the area of I/R-induced gastric lesions and the accompanying increase in the GBF as compared with the vehicle-pretreated control group (*p* < 0.05).

**Figure 4:**
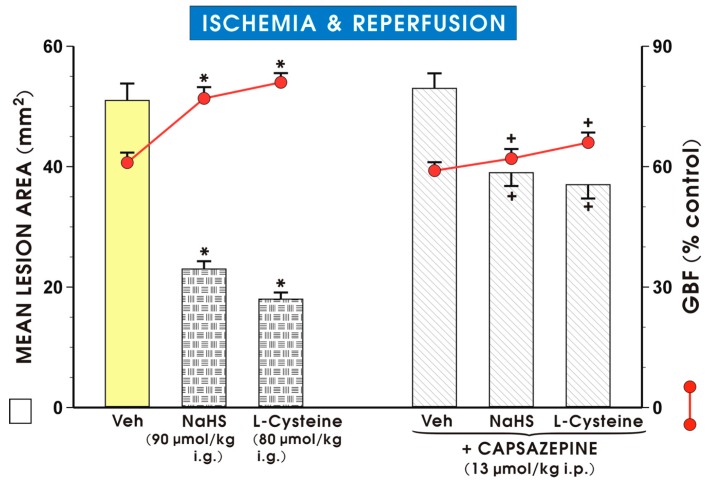
Mean lesion area and the GBF in rats pretreated with vehicle, NaHS (90 μmol/kg i.g.) or l-cysteine (80 μmol/kg i.g.) alone or in combination with capsazepine (13 μmol/kg i.p.) and exposed to I followed by 3 h of R. Results are mean ± S.E.M of 4–5 per each experimental group. Significant changes as compared with the respective values in the vehicle-control group are indicated by an asterisk (*p* < 0.05). A cross indicates a significant change compared to the values obtained in the group treated with NaHS and l-cysteine alone (*p* < 0.05).

Pretreatment with capsazepine (13 μmol/kg i.p.) which did not significantly increase the area of I/R-induced gastric damage and failed to significantly affect GBF, significantly increased the area of I/R-induced gastric lesions and significantly decreased the GBF evoked by NaHS or l-cysteine as compared with the groups treated with this H_2_S donor or H_2_S precursor alone (*p* < 0.05) (Figure 4).

**Figure 5:**
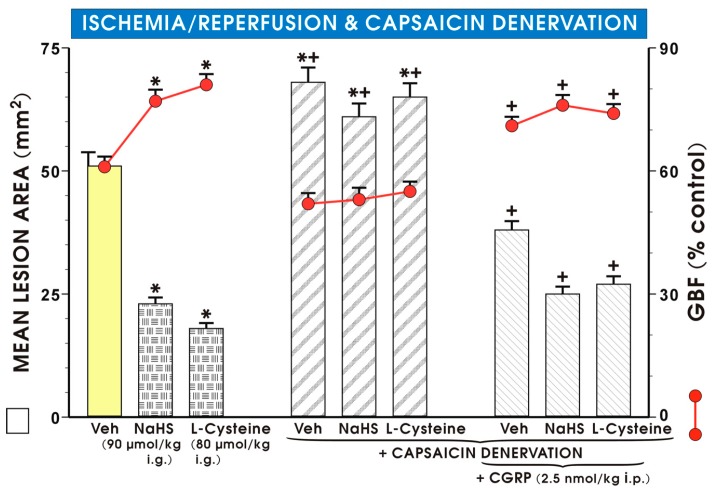
Effect of capsaicin denervation on the mean lesion area and GBF in rats exposed to 30 min of I followed by 3 h of R and pretreated with vehicle, NaHS (90 μmol/kg i.g.) or l-cysteine (80 μmol/kg i.g.) alone or in combination with CGRP (2.5 nmol/kg i.p.). Results are mean ± S.E.M of 4–5 rats per each experimental group. Significant change as compared with the respective values in vehicle-control group was indicated by asterisk (*p* < 0.05). Asterisks and crosses indicate significant changes compared to the values obtained in the group treated with saline, NaHS or l-cysteine without denervation (*p* < 0.05). A cross indicates a significant change as compared with the respective capsaicin-denervated animals without co-treatment with CGRP (*p* < 0.05).

In groups of rats with capsaicin-induced deactivation of afferent sensory nerves, the area of I/R gastric lesions was significantly increased and the GBF was significantly decreased after pretreatment with NaHS (90 μmol/kg i.g.) or l-cysteine (80 μmol/kg i.g.) as compared with rats with intact-sensory nerves pretreated with this H_2_S donor or its precursor (*p* < 0.05) (Figure 5). 

The combination of NaHS or l-cysteine with CGRP (2.5 nmol/kg i.p.) which by itself significantly reduced the I/R injury and significantly increased the GBF in rats with capsaicin denervation, restored the values of area of I/R-induced gastric lesions and the GBF to those observed in groups of rats pretreated with NaHS or l-cysteine alone without capsaicin denervation (Figure 5).

**Figure 6:**
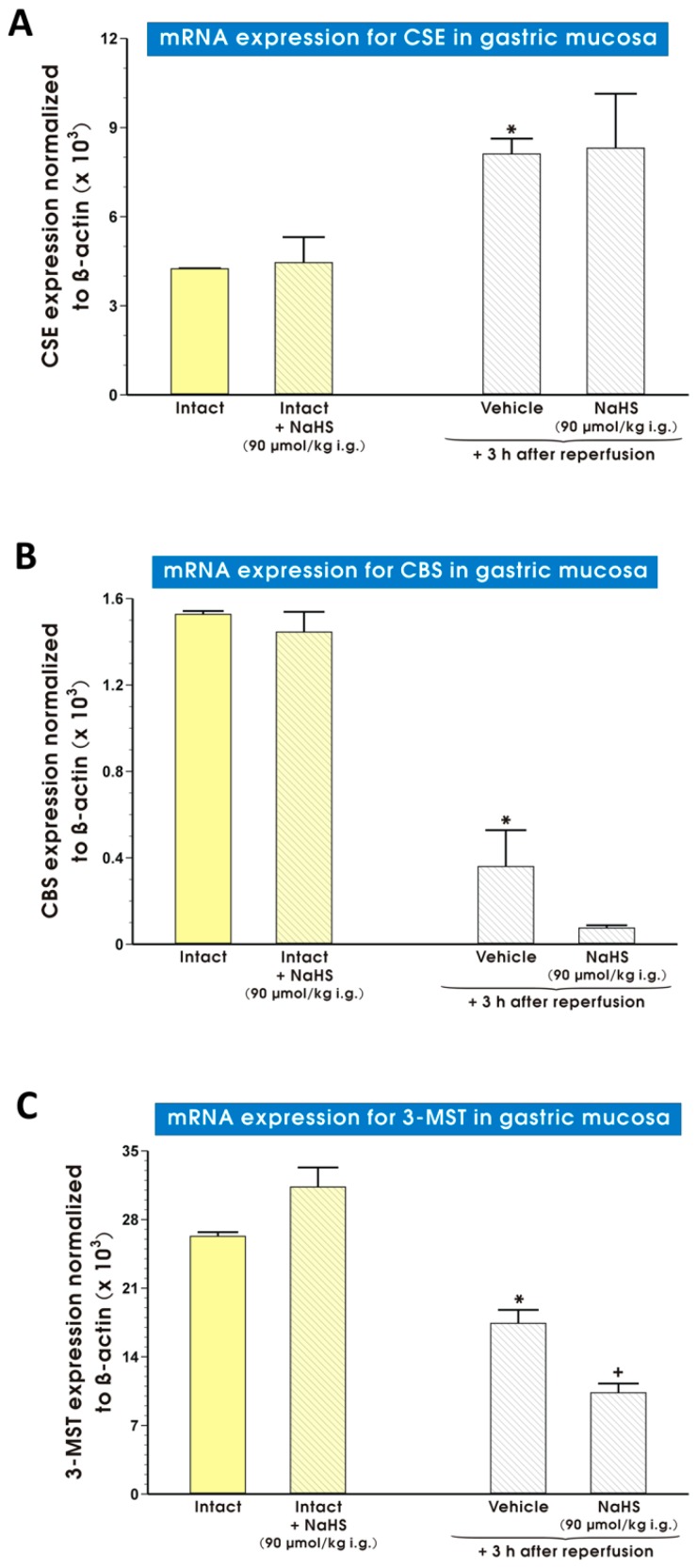
Expression of mRNA for CSE (**A**), CBS (**B**) and 3-MST (**C**) in gastric mucosa of rats pretreated with vehicle (saline) or NaHS (90 μmol/kg i.g.) with or without exposure to I followed by 3 h of R. Results are mean ± S.E.M of 4–5 rats per each experimental group. An asterisk indicates a significant change as compared with intact rats (*p* < 0.05). A cross indicates a significant difference as compared with vehicle pretreated group (*p* < 0.05).

Figure 6 shows the mRNA expression for CSE (Figure 6A), CBS (Figure 6B) and 3-MST (Figure 6C) in rats pretreated with vehicle or NaHS (90 μmol/kg i.g.) and exposed to I followed by 3h of R. In rats pretreated with vehicle or NaHS, a significant increase of CSE mRNA expression was observed after exposure to I/R (*p* < 0.05) (Figure 6A). In the groups pretreated with vehicle or NaHS, a significant decrease of mRNA expression for CBS (Figure 6B) and 3-MST (Figure 6C) was observed after I followed by 3 h of R (*p* < 0.05). NaHS (90 μmol/kg i.g.) administered to intact rats failed to affect mRNA expression for CSE (Figure 6A), CBS (Figure 6B) and 3-MST (Figure 6C) as compared with the group of rats treated with vehicle-saline instead of NaHS. 

**Figure 7:**
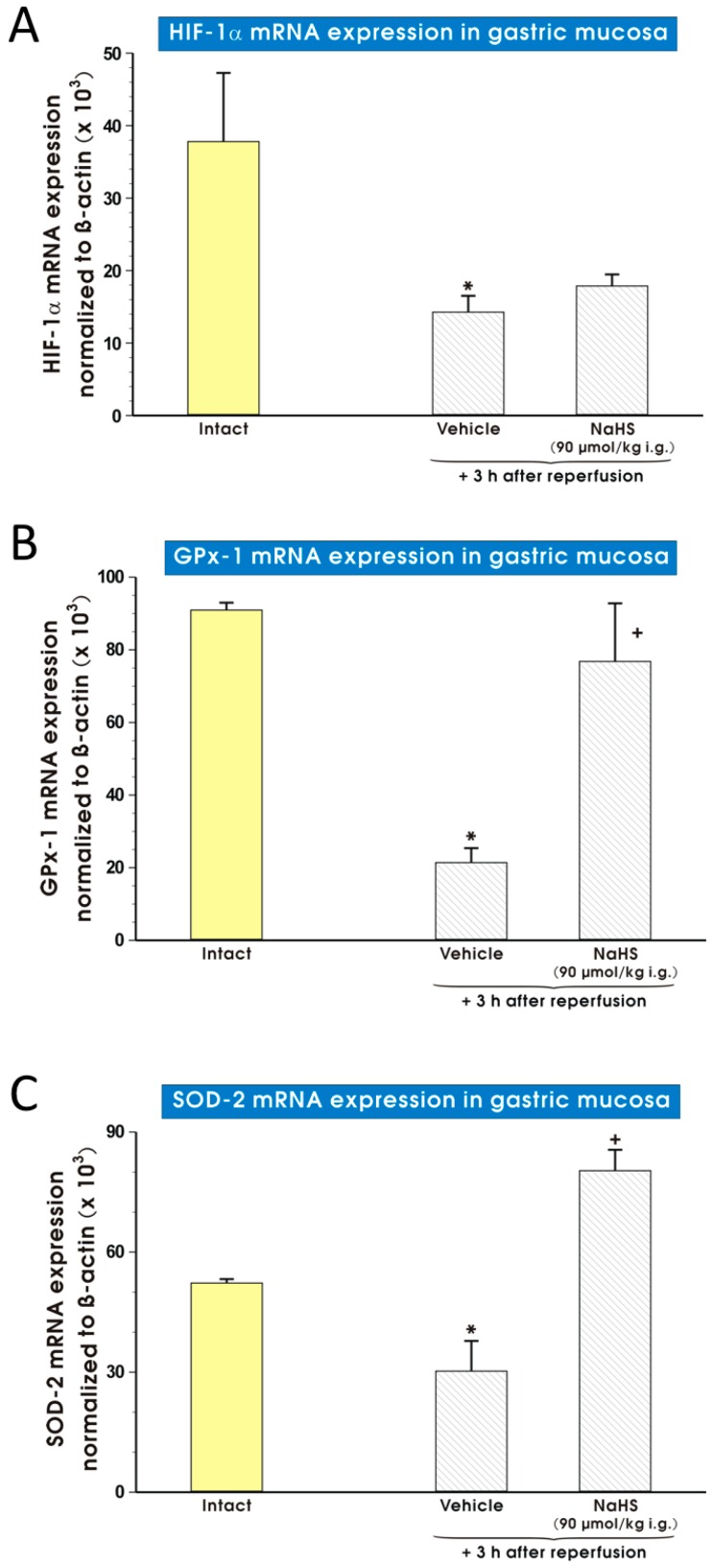
Expression of mRNA for HIF-1α (**A**), GPx-1 (**B**) and SOD-2 (**C**) in gastric mucosa of rats exposed to I followed by 3 h of R and pretreated with vehicle (saline) or NaHS (90 μmol/kg i.g.). Results are mean ± S.E.M of 4–5 rats per each experimental group. An asterisk indicates a significant change as compared with intact rats (*p* < 0.05). A cross indicates a significant difference as compared with the vehicle pretreated group (*p* < 0.05).

Figure 7 shows the mRNA expression for HIF-1α (Figure 7A), GPx-1 (Figure 7B) and SOD-2 (Figure 7C) in gastric mucosa of rats pretreated with vehicle or NaHS (90 μmol/kg i.g.) and exposed to I followed by 3 h of R. In rats pretreated with vehicle and exposed to I/R, the mRNA expression for HIF-1α, SOD-2 and GPx-1 was significantly decreased (*p* < 0.05) (Figure 7A–C). In groups pretreated with NaHS, the significant increase of GPx-1 (Figure 7B) and SOD-2 (Figure 7C) but not HIF-1α (Figure 7A) mRNA expression was observed after I followed by 3 h of R as compared with vehicle-control group (*p* < 0.05).

**Figure 8:**
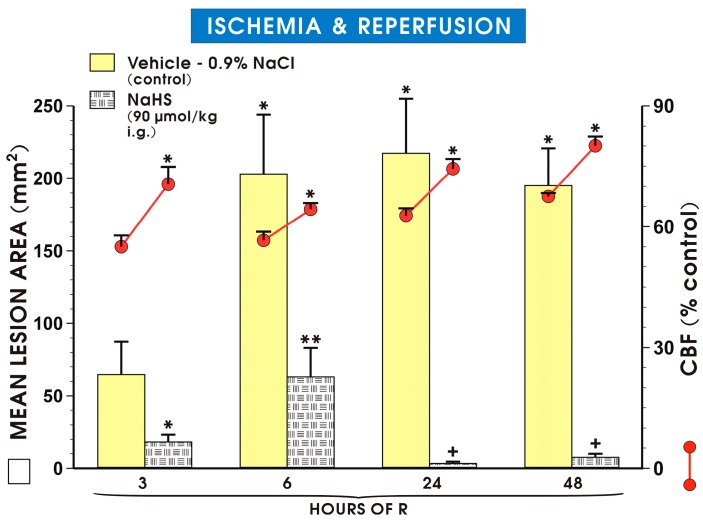
Mean lesion area and the GBF in gastric mucosa of rats pretreated with single dose of vehicle or NaHS (90 μmol/kg i.g.) and exposed to 30 min of I followed by 3, 6, 24 and 48 h of R. Results are mean ± S.E.M. of 4–5 rats per each group. An asterisk indicates a significant change as compared with the value obtained in the vehicle-control group exposed to I and 3 h of R (*p* < 0.05). Double asterisks denote a significant difference as compared with the values obtained in vehicle pretreated groups at respective times of R (*p* < 0.05). Crosses indicate a significant difference as compared with the values obtained in NaHS-pretreated groups at 3 h and 6 h of R, respectively (*p* < 0.05).

Figure 8 shows that gastric damage area was significantly increased in groups of vehicle-pretreated animals exposed to I followed by 6 h of R as compared with those exposed to I followed by 3 h of R and pretreated with vehicle or NaHS (90 μmol/kg i.g.) (*p* < 0.05).

Pretreatment with NaHS significantly decreased area of gastric I/R damage in groups exposed to I and followed by 3, 6, 24 or 48 h of R as compared to respective values recorded in vehicle-control group (*p* < 0.05). Pretreatment with NaHS accelerated the healing of I/R-induced gastric damage as demonstrated by a significant decrease in the area of gastric lesions in groups subjected to 24 and 48 h of R compared with the NaHS -pretreated groups exposed to 3 and 6 h of R (*p* < 0.05) (Figure 8).

**Figure 9:**
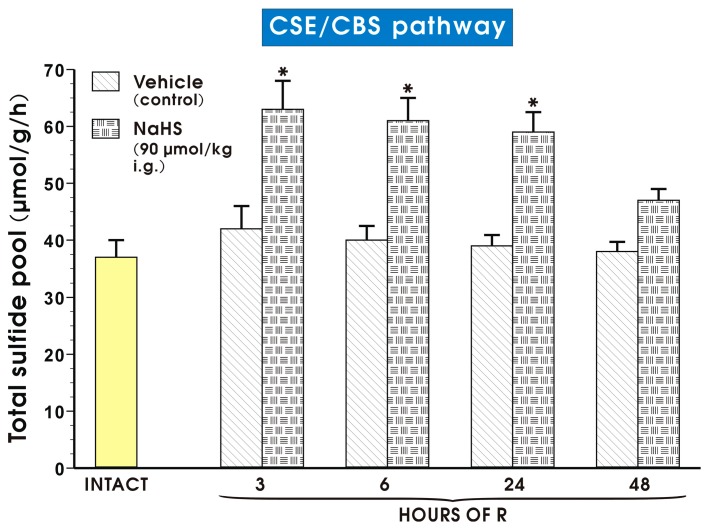
Total sulfide pool released by CSE/CBS activity pathway in gastric mucosa of intact rats and those pretreated with vehicle (saline) or NaHS (90 μmol/kg i.g.) and exposed to I followed by 3, 6, 24 or 48 h of R. Results are mean ± S.E.M of 4–5 rats per each experimental group. An asterisk indicates a significant change as compared with the value obtained in intact rats (*p* < 0.05).

Figure 9 shows that pretreatment with NaHS (90 μmol/kg i.g.) significantly increased sulfide release expressed as total sulfide pool from gastric mucosa exposed to I followed by 3, 6 and 24 h of R as compared with the time-respective vehicle-control groups (*p* < 0.05). In groups pretreated with vehicle or NaHS and exposed to I followed by 48 h of R, sulfide release reached the values similar to that obtained in intact rats.

**Figure 10:**
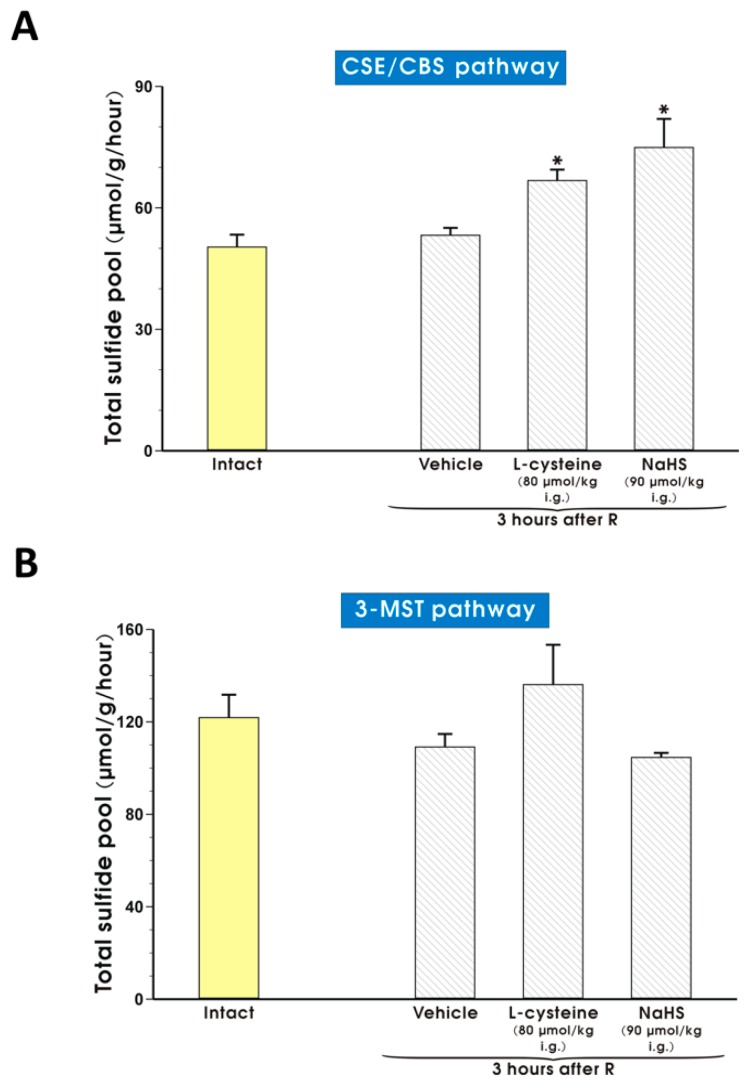
Total sulfide pool released by activity of CSE/CBS (**A**) or 3-MST (**B**) pathway from gastric mucosa of intact rats and those pretreated with vehicle (saline), l-cysteine (80 μmol/kg i.g.) or NaHS (90 μmol/kg i.g.) and exposed to I followed by 3 h of R. Results are mean ± S.E.M of 4–5 rats per each experimental group. Asterisk indicates a significant change as compared with the value obtained in intact rats (*p* < 0.05).

Figure 10 shows that pretreatment with l-cysteine (80 μmol/kg i.g.) or NaHS (90 μmol/kg i.g.) significantly increased sulfide release by the activity of CSE/CBS pathway in gastric mucosa exposed to I and followed by 3 of R as compared with vehicle-control group (*p* < 0.05) (Figure 10A).

Pretreatment with l-cysteine (80 μmol/kg i.g.) or NaHS (90 μmol/kg i.g.) did not affect sulfide release by the 3-MST activity pathway in gastric mucosa exposed to I and followed by 3 of R as compared with intact or vehicle-control group (*p* < 0.05) (Figure 10B).

## 3. Discussion

Exposure of gastric mucosa to I/R is known to induce gastric mucosal hemorrhagic lesions due to increased generation of reactive oxygen metabolites, microvascular dysfunction and adhesion of neutrophils to the vascular endothelium and their infiltration of gastric mucosal structure [24,25,26]. After the R, the reactive oxygen species are generated, especially from xanthine–xanthine oxidase system and due to activated neutrophils, leading to enhancement in tissue lipid peroxidation, which result in mucosal injury and cellular death [24,26,27]. As reported by our group previously, these acute I/R-induced gastric lesions can progress into deeper chronic ulcerations with the time extension of reperfusion [28]. 

In this study, we investigated the involvement of H_2_S, exogenously delivered by the administration of H_2_S donor, NaHS or slow H_2_S-releasing compound, GYY4137 [23] and that endogenously produced from l-cysteine, in the mechanism of gastroprotection against the formation of I/R-induced damage. Our second goal was to examine an effect of NaHS administration on further progression of these acute I/R lesions into deeper ulcerations by extension of the time of R from initial 3 h up to 6 h, 1 day and 2 days, respectively. We determined the possible role of H_2_S in restoration of gastric microcirculation and involvement of classic protective factors such as endogenous prostaglandins and afferent sensory nerves releasing vasodilatory mediators such as CGRP in H_2_S-mediated gastroprotection against I/R injury. We also attempted to assess if H_2_S released from NaHS can affect changes of mRNA expression for anti-oxidative HIF-1α, GPx-1 and SOD-2 induced by exposure of gastric mucosa to I/R.

We observed that pretreatment with NaHS, H_2_S donor or l-cysteine, which acts as a H_2_S precursor, dose-dependently reduced I/R-induced gastric damage and this effect was accompanied by restoration of gastric microcirculation. Similar effect was observed when animals were pretreated with GYY4137 which has been considered as a slow releaser of H_2_S. These results are in pair with previous data that the vasodilatory activity of H_2_S is involved in the mechanism of gastroprotection against stress-induced lesions [29]. Our results are also in keeping with previous observations that H_2_S exerted protective activity against I/R-induced gastric damage [21,22], hepatic I/R injury [30] and myocardial I/R injury [31,32]. In addition, Meng et al. [33] reported that GYY4137 protects against myocardial I/R injury. In this study, we have observed that CSE but not CBS or 3-MST mRNA expression in gastric mucosa was elevated after exposure to I/R suggesting that in rat stomach injured by I/R, the H_2_S can mainly derive from the activity of CSE biosynthetic pathway. Moreover, pretreatment with NaHS or l-cysteine increased endogenous H_2_S production, possibly due to CSE activation since sulfide release from gastric mucosa exposed to I/R and pretreated with NaHS or l-cysteine was increased via CSE/CBS but not 3-MST activity pathway. Similarly, Bos et al. [34] demonstrated that CSE activity could be considered as protective enzymatic pathway against renal damage caused by I/R.

Based on the evidence presented in this study, we propose that H_2_S gastroprotection against I/R-induced injury does not depend on endogenous PGs biosynthesis by COX-1 or COX-2 activity since the pharmacological inhibition of the activity of these enzymes failed to affect beneficial effect of NaHS or l-cysteine observed in our present study. This seems to be contrary to the previous observation in another experimental model of gastric damage because NaHS increased COX-1, COX-2 and PGE_2_ content and prevented gastric mucosa against stress-induced lesions [29]. Wallace et al. [35] demonstrated that decreased H_2_S biosynthesis in rodent colon is associated with downregulation of COX-2 mRNA expression. We assume that these discrepancies can be explained by the evident differences in animal models and experimental conditions used in our and previous studies. 

The mechanism of H_2_S-induced protection against I/R-injury involves the anti-oxidative properties of this gaseous molecule since in our study the pretreatment with NaHS increased mRNA expression for SOD-2 and GPx-1. On the other hand, in gastric mucosa compromised by I followed by 3 h of R, HIF-1α mRNA expression was also decreased. Indeed, previous studies reported that mRNA expression of the gene for this particular protein was not affected in gastric mucosa when stomach was subjected to standard I/R injury with an extension of reperfusion even up to 1 or 6 days [36]. In contrary, Wang et al. [37] reported that HIF-1α protein expression was increased in gastric mucosa compromised by I and 6 h of R, suggesting that expression of this hypoxic factor in gastric mucosa could be time of reperfusion-dependent. Our study revealed that pretreatment with NaHS failed to affect downregulation of HIF-1α mRNA expression in gastric mucosa after standard 3 h of R. Therefore, we conclude that H_2_S-mediated gastroprotection against this type of injury does not involve regulation by HIF-1α.

On the other hand, we observed that, the gastroprotective effect of NaHS and l-cysteine was attenuated in rats with capsaicin-induced sensory nerves ablation. The NaHS- and l-cysteine-mediated gastroprotection in capsaicin-treated rats was then restored when CGRP was co-administered with H_2_S donor or its metabolic precursor. Similarly to capsaicin-denervation, inhibition of TRPV1 activity by capsazepine [38,39], although not completely, reversed gastroprotective effect of NaHS and l-cysteine. Therefore, we assume that the mechanism of H_2_S-mediated regulation of gastric microcirculation and gastroprotection against I/R-induced damage may depend upon the activation of TRPV1 receptors and the activity of afferent sensory nerves releasing vasodilatory CGRP. Based on these results obtained in rats with capsaicin denervation and those with inhibited TRPV1 receptors by capsazepine, we conclude that the potent vasodilatory and gastroprotective activity of H_2_S against I/R gastric lesions may depend, at least in part, on activation by this gaseous molecule of sensory nerves and vanilloid receptors. Since some degree of H_2_S protection was still observed in rats pretreated with capsazepine, the possibility is not excluded that other factors i.e., nitric oxide (NO), potassium channels, guanylyl cyclase/cGMP system, or yet unidentified factors can also mediate this protection and an increase in the GBF observed in rats pretreated with H_2_S donor. Our notion that H_2_S protection involves sensory nerves activation matches with data published recently that H_2_S-induced protection against gastric oxidative bleeding erosions caused by another ulcerogen such as stress has been ameliorated in animals with capsaicin-denervation [29]. Indeed, Lu et al. [40] provided direct evidence that H_2_S modulates intestinal motility and excites duodenal contraction by the mechanism involving activation of TRPV1 in afferent nerves endings and opening of K_ATP_ channels.

Interestingly, we observed that pretreatment with NaHS not only protected gastric mucosa against the formation of I/R-induced lesions but also reduced development of gastric damage during their progression to deeper gastric ulcerations and restored the decrease in GBF as observed after 6 h of R in vehicle-pretreated animals exposed to I/R. Moreover, in groups pretreated with NaHS, almost no gastric damage after 24 and 48 h of R has been observed. Furthermore, the exposure to I/R resulted in upregulation of mRNA expression for CSE but downregulated CBS and 3-MST mRNA expression in gastric mucosa and this effect was not affected by NaHS. Additionally, endogenous production of H_2_S in gastric mucosa has been increased in rats pretreated with donor of this gaseous mediator. We assume that elevated H_2_S bioavailability caused by the administration of NaHS releasing H_2_S enhanced gastric mucosal resistance to oxidative I/R-induced gastric mucosal damage. Moreover, H_2_S production in gastric tissue was maintained after 6 and 24 h of R. Thus, we conclude that H_2_S can strengthen the mucosal barrier since this gaseous mediator not only reduced I/R-induced gastric damage formation but also accelerated healing of these acute erosions progressing with time into gastric ulceration. This supports the observation by Wallace et al. [17], who reported that treatment with H_2_S donors accelerated the healing of pre-existing gastric ulcers.

## 4. Materials and Methods

### 4.1. Animals

Male Wistar rats (*n*= 150, average weight about 250 g) were used in the study. Rats were fasted for 24 h with free access to drinking water before the exposure to I/R. The study was approved by the Institutional Animal Care and Use Committee of Jagiellonian University Medical College in Cracow and run in accordance with the statements of the Helsinki Declaration regarding handling of experimental animals.

### 4.2. Experimental Design, I/R Injury, Afferent Sensory Nerves Ablation

Acute I/R gastric lesions were induced as described previously [28,41,42]. Briefly, under pentobarbital anesthesia (60 mg/kg i.p.), the abdomen was opened, and the celiac artery was identified and clamped for 30 min (I) followed by removal of the clamp to obtain R. After 3, 6, 24 and 48 h of R, the area of gastric damage was measured by planimetry and the functional parameters described below were determined. 

Rats with intact sensory nerves (series A) were randomized to the groups (4–5 rats each) and were administered 30 min before exposure to I/R with: (1) saline (vehicle-control group); (2) NaHS (18–180 μmol/kg i.g.); (3) l-cysteine (0.8–80 μmol/kg i.g.); (4) GYY4137 (90 μmol/kg i.g.). The doses of 90 and 80 μmol/kg i.g. for NaHS and l-cysteine, respectively, have been selected based on ability of these compounds to reduce the area of I/R gastric damage by 50%. Separate groups of rats were treated with saline or NaHS (90 μmol/kg i.g.) and were not exposed to I/R. 

Rats of series B were pretreated with capsazepine (5 mg/kg i.p.), TRPV1 blocker [38,39], indomethacin (14 μmol/kg i.g.), non-selective COX inhibitor, SC-560 (14 μmol/kg i.g.), selective COX-1 inhibitor or celecoxib (26 μmol/kg i.g.), selective COX-2 inhibitor alone or in combination with NaHS (90 μmol/kg i.g.) or l-cysteine (80 μmol/kg i.g.) [29,43].

Two weeks before experiment, in separate group of rats (series C), capsaicin was administered for three days at a dose of 82.5, 165 and 165 μmol/kg (total dose: 412.5 μmol/kg s.c.) to induce functional ablation of afferent sensory nerves as described previously [42,44]. Next, rats of series C were pretreated with vehicle, NaHS (90 μmol/kg i.g.) or l-cysteine (80 μmol/kg i.g.) alone or in combination with CGRP (2.5 nmol/kg i.p.) and exposed to I/R.

In series D, rats were pretreated with vehicle or NaHS (90 μmol/kg i.g.) and were exposed to I followed by 3, 6, 24 and 48 h of R to investigate the progression of I/R injury and changes in H_2_S production in gastric mucosa. All tested compounds were of analytical grade and were purchased from Sigma-Aldrich (Schelldorf, Germany).

### 4.3. Determination of I/R Lesions Area and Gastric Blood Flow Level (GBF)

After the end of I/R, the animals of series A–D were anesthetized with pentobarbital (60 mg/kg i.p.), the abdomen was opened, and the stomach was exposed to measure GBF by H_2_ gas clearance technique as described previously [45]. The GBF was measured in the fundic part of the gastric mucosa not involving mucosal lesions. Average values of three measurements were determined and expressed as a percentage of the change of the value determined in intact gastric mucosa of rat stomach. The area of gastric lesions was determined with computerized planimetry (Morphomat, Carl Zeiss, Berlin, Germany) by a person who did not know which experimental group the animals belonged to.

### 4.4. Measurement of Sulfide Production in Gastric mucosa

Measurement of sulfide production in gastric mucosa was determined by modified methylene blue-method, as described previously [17,19,29]. Briefly, gastric mucosa was quickly isolated, snap-frozen, and stored at −80°C. The gastric tissue was homogenized in ice-cold 50 mM potassium phosphate buffer, pH = 8.0. l-cysteine (10 mM) and pyridoxal 5′-phosphate (P5P; 2 mM) or α-ketoglutarate (α-KG; 2 mM) were added. H_2_S biosynthesis via CSE and CBS required the presence of P5P, while that via 3-MST required α-KG. Tube containing a piece of filter paper soaked with zinc acetate (1%; 0.3 mL) was placed inside the larger vial. The vials were then flushed with nitrogen gas and capped with an airtight serum cap. The vials were then transferred to water bath (37 °C) and after 90 min, trichloroacetic acid (TCA; 50%; 0.5 mL was injected into the reaction mixture through the serum cap. The mixture remained to stand for 60 min. *N*,*N*-dimethyl-*p*-phenylenediamine sulfate (20 mM; 50 μL) in 7.2 M HCl and FeCl_3_ (30 mM; 50 μL) in 1.2 M HCl were added. After 20 min, absorbance at 670 nm was measured with a microplate reader (Tecan Sunrise, Männedorf, Switzerland). The calibration curve of absorbance vs. H_2_S concentration was obtained by using NaHS solution of varying concentrations. Results were expressed as total sulfide pool released from 1 g of gastric mucosa per one hour.

### 4.5. Determination of mRNA Expression for CSE, CBS, 3-MST, HIF-1α, GPx-1, SOD-2 in Gastric Mucosa by Real Time Polymerase Chain Reaction (PCR)

Gastric mucosa from rats exposed to I/R and pretreated with vehicle or NaHS (5 mg/kg i.g.) was collected, snap-frozen in liquid nitrogen and stored at −80°C for further determination of mRNA expression for CSE, CBS, MST, HIF-1α, GPx-1, SOD-2 by real time PCR, as described previously [18,19,46].

Total RNA was isolated using commercially available kit with spin-columns (GeneMATRIX Universal RNA Purification Kit, EURx, Gdansk, Poland) according to manufacturer protocol. Reversed transcription was performed using High-Capacity cDNA Reverse Transcription Kit with RNAse inhibitor (MultiScribe™, Applied Biosystems, Life Technologies, Carlsbad, CA, USA) and random primers. 

Expression for CSE, CBS, MST, HIF-1α, GPx-1, SOD-2 and β-actin as reference gene was determined using specific primers [18,19]. The real time PCR was conducted using thermal cycler Quant Studio 12K Flex (Thermo Fisher Scientific, Waltham, MA, USA) and SYBR Green dye including kit (SG qPCR Master Mix (2x), EURx). Results were analyzed using the 2^−ΔCt^ method [47,48].

### 4.6. Statistical Analysis

Results of the experiment were expressed as mean ± S.E.M and the statistical analysis was performed with t-Student test or ANOVA test and Tukey *post-hoc* test where appropriate. *p* < 0.05 was considered as statistically significant.

## 5. Conclusions

We conclude that H_2_S released form NaHS or produced endogenously form l-cysteine mainly due to CSE activity acts as an important, protective factor in gastric mucosa against I/R-induced gastric damage which can also accelerate the healing of these erosions. The mechanism of this H_2_S-mediated gastroprotection does not involve the endogenous PGs biosynthesis and regulation of HIF-1α expression but may depend upon the vasodilatory properties of this gaseous molecule mediated by the activity of CGRP-releasing afferent sensory nerves, finally leading to increase in gastric microcirculation under conditions associated with the generation of reactive oxygen metabolites and the enhanced oxidative metabolism.
